# Naturally occurring genotype 2b/1a hepatitis C virus in the United States

**DOI:** 10.1186/1743-422X-8-458

**Published:** 2011-10-03

**Authors:** Dipankar Bhattacharya, Molly A Accola, Israr H Ansari, Rob Striker, William M Rehrauer

**Affiliations:** 1 University of Wisconsin-Madison, School of Medicine and Public Health, Department of Medicine, Madison WI 53706 USA; 2 University of Wisconsin Hospitals and Clinics, Clinical Laboratories, Highland Avenue, Madison WI 53792 USA; 3 W. S. Middleton Memorial Veteran's Association Hospital, 2500 Overlook Terrace, Madison, WI 53705 USA; 4 University of Wisconsin-Madison, School of Medicine and Public Health, Department of Pathology and Laboratory Medicine, Madison WI 53792 USA

## Abstract

**Background:**

Hepatitis C Virus (HCV) infected patients are frequently repeatedly exposed to the virus, but very few recombinants between two genotypes have been reported.

**Findings:**

We describe the discovery of an HCV recombinant using a method developed in a United States clinical lab for HCV genotyping that employs sequencing of both 5' and 3' portions of the HCV genome. Over twelve months, 133 consecutive isolates were analyzed, and a virus from one patient was found with discordant 5' and 3' sequences suggesting it was a genotype 2b/1a recombinant. We ruled out a mixed infection and mapped a recombination point near the NS2/3 cleavage site.

**Conclusions:**

This unique HCV recombinant virus described shares some features with other recombinant viruses although it is the only reported recombinant of a genotype 2 with a subtype 1a. This recombinant represents a conundrum for current clinical treatment guidelines, including treatment with protease inhibitors. This recombinant is also challenging to detect by the most commonly employed methods of genotyping that are directed primarily at the 5' structural portion of the HCV genome.

## Background

The WHO estimates that 130-170 million people worldwide are infected with HCV [1]. Six major genotypes (lineages) of HCV have spread throughout the world [2,3]. Viral genotype is well recognized as the most significant prognostic factor in terms of response to therapy, and a characteristic upon which to base the antiviral prescription [4]. Therefore, the best methods and the most definitive viral target(s) for determining the HCV genotype during patient care remains an important area of translational research. Infection with any genotype can lead to liver cirrhosis and liver cancer in a minority of patients. While specific genotypes dominate in certain regions of the world (for example, genotype 4 in the Middle East), many regions have multiple genotypes circulating including Europe and the United States. People with repeated use of intravenous needles and contaminated blood products in these regions likely are exposed to more than one genotype of HCV. Both mixed infections and recombinant viruses have been described. These situations are thought to be uncommon, particularly natural recombination between two genotypes. Genetic incompatibilities between the viral proteins of different genotypes have been suggested as a reason for recombination in HCV to be a rare event [5].

Here we describe a case report of a patient who was chronically infected over an extended period of time with a recombinant HCV strain. Like all other naturally occurring inter-genotypic recombinants reported to date, this strain has a genotype 2 5' portion of the genome encoding the structural region, while the nonstructural coding region is from a different genotype. The crossover junction was mapped to the NS2/3 region. We also compared the sequence to other recombinants, but could find limited evidence to support the proposed theory that stable RNA hairpin structures can promote recombination and bracket the cross over junction.

## Methods

### Clinical Genotyping

Total nucleic acid was extracted from patient plasma using the Roche AmpliPrep^® ^TNAI kit (Roche, Indianapolis, IN). The nucleic acid was reverse-transcribed with random primers, AMV RT, and 0.2 mM deoxynucleotides (Promega, Madison, WI). PCR was performed on a Roche LightCycler^® ^480 with Roche LightCycler^® ^480 SYBR Green I Master mix, primers (A and B in Table 1; [6-8]) specific for either the 5'UTR or NS5B regions (TIB Molbiol, Adelphia, NJ) and 2uL of the RT-PCR product. Products of amplification were sequenced bidirectionally using Big Dye (Applied Biosystems) on an Applied Biosystems 3100 instrument. Sequences were compared to a database consisting of 100 reference HCV 5'UTR and 58 reference NS5B sequences from the Los Alamos National Laboratory [9] using Assign-ATF software (Conexio Genomics, Fremantle, Australia).

**Table 1:** Primers used for Amplification and DNA Sequencing of 5' UTR and NS5B regions in the identification of the HCV recombinant isolate (A, B) and subsequently for Amplification of Overlapping Products Across Entire HCV Recombinant Genome (1-7)

Amplicon	Nucleotide Number (H77 numbering Accession no. AF009606)	Forward Primer (5'-3') Normal: HCV genotyping primer; **Bold: 1a primer;***Italics: 2b primer *	Reverse Primer (5'-3') Normal: HCV genotyping primer; **Bold: 1a primer;***Italics: 2b primer*
A	73-313	AAGCGTCTAGCCATGGCGT [7]	CACTCGCAAGCACCCTATCA [6]
B	8256-8644	TATGAYACCCGCTGYTTTGACTC [8]	GCNGARTAYCTVGTCATAGCCTC [8]
1	92-1295	*TAGTATGAGTGTCGTACAGCCTCC*	**TAGGGGACCAGTTCATCATCATATCCCATGCC**
			*ATGACTTTGGCCCACGCTCCCTGCATGG*
2	1171-2240	*TGGGAGACGTGTGTGGGGCCGTGATGATCGTGTCGCAGGC*	**TTGGTATATCCAGTTGAGTTCATCCAGGTGCAACCGAACC**
			*TGCGGACAATCGATGCTCCACCCCCCCTACATACATCCG*
3	2108-2980	*TTTTAGGAAGCACCCAGATACCACCTATCTTAAGTGTGGAGC*	**TTGCGCTTCTGCTCTAGTCAGAAAATACTGAAGCCACC**
			*TGACCTCAAACACAAGGCGTGGGTGTAGAATGACAGCC*
4	2901-4793	*TTAGTCTTGGCCGAGGCCCAGATTCAGCAATGGG*	**TGGTTGTCTCAATGGTGAAGGTAGGGTCAAGGC**
			*TTCTCCCTCTACGTTGACTACGGGAGACAGC*
5	4707-5789	**ATAGACTGTAACACGTGTGTCACCC**	**AGGACCTTCCCCAGTCCAACAC**
6	5758-7285	**CCACTGGCCAAACCCTCCTCTTCAACATA**	**ACCACAGGTGGTTCGTAGTCGGGCTTTTTCCACGCC**
7	7218-9296	**GCGCGGCCGGACTACAACCCCCCGCTGATAGAGGCGTGG**	**TTCATTATGAAAGGATCCGCGGGGTCGGGCACGAGACAGGCTGTG**

### Sequencing of Recombinant

**Figure 1:**
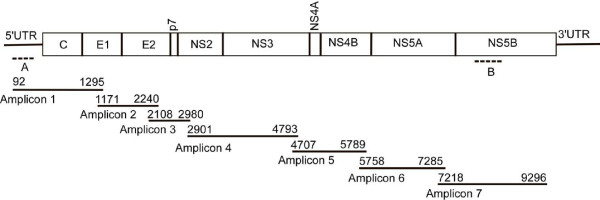
**Schematic of amplification products used to genotype discordant virus and to sequence recombinant virus**. Regions of the HCV 5'UTR (A) and NS5B (B) were amplified on all patients at UWHC for a 12 month period of time and sequenced using primers in Table 1. Amplification products 1-7 were generated and sequenced separately using primers listed in Table 1.

Seven different fragments (Figure 1, Parts of HCV genome 1-7) of the discordant virus identified were sequenced. Each fragment of cDNA synthesis was done from viral RNA, extracted from patient serum, by SuperScript III One-Step RT-PCR System with Platinum Taq DNA Polymerase (Invitrogen, CA) kit according to the manufacturer's protocol. Forward and reverse primers were designed based on an alignment [10] (ClustalW program available online from http://www.genome.jp/tools/clustalw/) of 217 genotype 1a sequences and 26 genotype 2 sequences from the European HCV databases [11]. Each primer from the alignment was selected from regions displaying 40-70% consensus within the respective alignment. RT-PCR amplification was performed by adding an entirely conserved forward primer from the recombinant virus and both 1a and 2b reverse primers from the alignment into the reaction (Table 1) to generate amplification products 1-4 (Figure 1). Fragments 5, 6, and 7 were done only with 1a primers. PCR fragments were restricted to be between 900 to 2000 basepairs. Seven fragments were amplified from RNA covering the full polyprotein and the 5' UTR of the recombinant virus. After amplification the PCR products were gel purified and cloned into a TOPO TA cloning vector (Invitrogen, CA) according to the manufacturer's protocol. After identifying the positive clones the cDNA inserts were sequenced bidirectionally with Big Dye (Applied Biosystems) at the UW-Madison Biotechnology facility.

### Bioinformatic Analysis

A web-based program http://www.phylogeny.fr/ was used to analyze the recombinant strain relative to other HCV strains [12]. All sequencing data were collected and searched against the NCBI database using a web-based nucleotide BLAST program http://blast.ncbi.nlm.nih.gov/Blast.cgi. For identification of a more specific junction, analyses were performed using the SimPlot program [(Version 3.5.1) [13] available online from http://sray.med.som.jhmi.edu/SCRoftware/simplot]. The RNA secondary structure was analyzed using the MFOLD program with default parameters. The MFOLD web based program is provided by Michael Zuker, Rensselaer Polytechnic Institute http://mfold.rna.albany.edu/?q=mfold/RNA-Folding-Form. The stable RNA secondary structure for this chimeric virus was analyzed and compared with the stable hairpin structure 1 as described by [14]. The NS2-NS3 region near the crossover point was also analyzed using the same program.

### GenBank nucleotide sequence accession number

The entire nucleotide sequences of this recombinant virus have been submitted to GenBank and assigned accession number is JF779679.

## Results

### DNA Sequencing clinical isolates at two different regions of the HCV genome produces mostly concordant results

**Figure 2:**
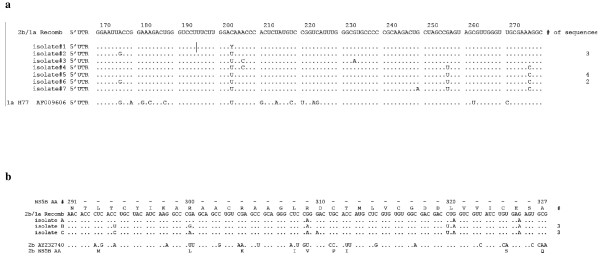
**Fewer unique sequences of HCV subtype 2b (Panel A) than 1a (Panel B) were identified at UWHC**. A) DNA sequence of the 5' UTR of the discordant 2b/1a strain is aligned with several representative non-discordant 2b strains, as well as the 5' UTR of the prototypical 1a H77 strain. These 8 sequences represent all the sequence diversity of this region of the 14 2b subtypes found in the 12 months in which both the 5' UTR and NS5B regions were sequenced. B) Alignment of the NS5B protein and nucleotide sequence of the 2b/1a discordant strain with three typical 1a strains as well as a 2b reference strain. NS5B DNA sequence of isolate A is quite close to the 2b/1a strain, but the NS5B DNA sequences of isolates B and C are less similar, yet more typical of 1a strains from UWHC, with 3 identical sequences from the 90 subtype 1a sequences determined in the 12 month period.

Over a 12 month period, the Molecular Diagnostic lab at the University of Wisconsin Hospital and Clinics (UWHC) performed a bidirectional consensus sequencing reaction of 203 base pairs of the 5' UTR as well as a separate bidirectional consensus sequencing reaction of the 222 base pair fragment of NS5B for 133 consecutive patients. As expected, based on genotype 1 being the most common in the United States, almost 70% of the time the NS5B subtype was 1a (90 different patients). Therefore, if a patient in our population was exposed to multiple HCV strains both the first and the second exposure might be subtype 1a viruses. In all cases but one, the genotype determined by the 5' UTR matched that determined by the NS5B fragment (subtype discrepancies between genotype 1 were unresolvable due to the lack of specificity of the 5 'UTR [15,16]. The only exception was an isolate with a 5' UTR that matched best with genotype 2b sequences (Figure 2a), while the corresponding NS5B sequence from that isolate matched best with a genotype 1a sequence (Figure 2b). Neither the 5' UTR 2b sequence nor the NS5B sequence perfectly matched with previously determined sequences in our patient population during this study period, although the 2b sequence was identical to an isolate from one patient prior to this twelve month period of time which had been identified as a 2b 5' UTR (see Figure 2a). Sequencing of the isolate with the near identical 2b 5' UTR sequence by NS5B primers was consistent with a 2b genotype. Furthermore the uridine position at base pair 204 of the 5' UTR (numbering according to H77 GenBank # AF009606) from this isolate was found to be a mixed population containing both uridine as well as the cytidine more commonly seen among isolates characterized as 2b at our institution.

### The discordant clinical isolate is a 2b/1a recombinant virus with a crossover at the NS2/NS3 Junction

**Figure 3:**
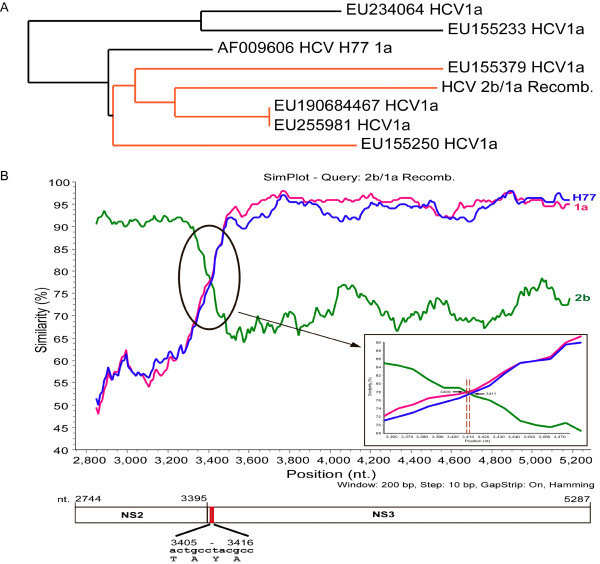
**The HCV recombinant 2b/1a isolate crosses over near the NS2/NS3 junction**. A) Phylogeny of NS3-NS5B black lines represent accession numbers previously identified as clade 1a1, and red lines represent accession numbers identified as 1a2 [17]. B) Simplot of 2b/1a recombinant referenced against a subtype 1a clade 1 (H77), a subtype 1a clade 2 strain and a subtype 2b reference strain.

Since isolates from four separate blood draws of a single patient over 5 months yielded a discordant 2b and 1a genotype from DNA sequencing of the 5' UTR and NS5B regions, respectively, both subtype specific 2b and 1a primers were designed and used to amplify from base pair 73-313 and 8256-8644 (Table 1, Figure 1) across the HCV genome. Strong amplification was seen with 2b primers throughout the structural regions, but not downstream of NS2. Conversely, 1a primers either failed or weakly amplified the structural gene region upstream of NS2, but strongly amplified sequences encoding the nonstructural proteins. The entire reconstructed genome was submitted to GenBank (accession # JF779679 hereafter denoted RF8_2/1a). Previously, we and others have noted that subtype 1a has two distinct clades provisionally termed 1a1 and 1a2 [17]. Both clades are widely dispersed temporally and across the globe with the prototypical H77 strain being representative of the 1a1 clade. Phylogenetic analysis of just the NS3-5B region of this recombinant strain shows it partitions with strains in the 1a2 clade (Figure 3A) rather than strains in the 1a1 clade including H77. A representative 2b genome (AY232740), the H77 genome (AF009606), as well as a 1a2 genome (EU255981) were all used as reference sequences to compare with the recombinant sequence via Simplot. As shown in Figure 3B, the identity of this strain is over 95% with a 2b sequence (AY232740) until the end of the NS2 gene, at which point it drops off below 82%, but climbs to over 95% identity when compared to a genotype 1a strain (EU255981). Most breakthrough viremia in patients on protease inhibitors that has been characterized to date has occurred in subtype 1a infected patients [18]. This is consistent with a codon bias between 1b and 1a isolates in which most 1b patients require two mutations to occur in the codon for residue 155 of NS3, whereas most 1a patients require only a single base pair change to encode for lysine rather than arginine. Accordingly, the NS3 sequence from this recombinant isolate also requires only a single base pair change from UUG to encode a lysine and subsequently confer protease resistance.

While HCV recombinants between two different genotypes are rare, closely related putative parental strains and several different isolates of the RF_2k/1b have been sequenced. Based on RNA structural analysis and recombinants seen in the plant virus turnip crinkle virus, mechanisms that might lead to recombination have been proposed including the identification of two stable RNA hairpins upstream and downstream of the crossover site in the parental strains that are putatively destabilized by mutations in the recombinant [14]. For example, the hairpin structure 1 (HS1) observed in the recombinant is present in the parental 2k strain, but is destabilized slightly by the acquisition of two mutations present in the recombinant RF1_2k/1b. Additionally, a hairpin downstream of the crossover site in the 1b parental strain was destabilized by 5 mutations present in the RF_2k/1b recombinant. We examined if this hairpin was also present in the RF8_ 2/1a reported here or in any of the other recombinants with a genotype 2 5' UTR-NS2 region. Although, all recombinants exhibited RNA base pairing in this region, all secondary structures appeared less stable than that of RF_2k/1b (data not shown), with two small stems being predicted rather than one longer stem. Non-recombinant 2b and the RF8_ 2/1a have similarly stable hairpins in this region, however, neither are as stable as the 2k hairpin (recombinant or parental).

## Discussion

For over ten years, HCV genotyping has been the critical parameter to determine both the likelihood of response to therapy, as well as the duration of therapy needed to obtain a Sustained Virologic Response (SVR) [19-21]. While several HCV genotyping methods exist, none are FDA approved in the United States. These methods are based on primarily targeting only the 5'structural regions of virus and thus cannot easily identify recombinant strains. Based on the last Hepatitis Viral Load proficiency survey (HVL-C) administered by the College of American Pathologists in 2010 [22], the most commonly employed HCV genotyping method, utilized by over 60% (110/177) of participating clinical diagnostic labs in the United States, is the Versant HCV (LiPA) 2.0 assay (manufactured by Innogenetics, distributed by Siemens Healthcare Diagnostics). While CE marked in Europe and approved for research use only in the United States, this assay is based on line probe hybridization targeting DNA sequence information within the 5' UTR and the contiguous structural core (C) region of HCV (Figure 1). While 9% (16/177) of participants did conduct genotyping by DNA sequencing using the Siemens Diagnostics TruGene system, sequencing information is again only obtained from the 5' UTR of HCV. These 5' structural regions have been utilized historically for genotyping because they are adequately conserved such that a limited number of primers or probes can amplify and recognize all isolates, respectively, but have sufficient diversity to distinguish between non-recombinant genotypes 1-6. However, it should be noted that information provided solely by the 5'UTR is insufficient for subtype identification and in some cases for genotype identification.

**Figure 4:**
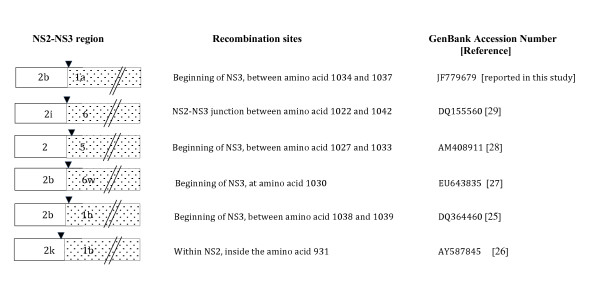
**Comparison of genotypic HCV recombinants previously reported**. All full-length recombinants described with a single recombination point include a genotype 2 5' UTR. Only the 2k/1b recombinant has been identified in multiple patients.

Several studies looking for HCV recombinants in intravenous drug users and other populations where multiple exposures are likely have been performed and in general recombination does seem to be a rare event [23]. While other reports of recombination between different genotypes exist [24], the DNA sequence of entire recombinant genomes and site of recombination remains to be determined. Interestingly, all full-length recombinants described to date between two genotypes have included a 5' portion of genotype 2 [25-29]. So far only RF1_2k/1b has been shown to be circulating in multiple patients described in Russia [26], Ireland [30], Estonia [31] and Uzbekistan [32]. As shown in Figure 4, all reported HCV recombinants have similar, but non-identical cross over points to the RF8_2/1a (reported here) or RF1_2k/1b.

It remains unclear how much of the genome needs to be genotype 2 in order for the clinical response to justify a 12-24 week treatment course rather than 48 weeks advocated for genotype 1. Both within genotype 1, as well as between genotypes 1 and 2, there are known differences between the ability of NS5A to bind the ds RNA induced PKR [33]. These differences in NS5A binding alter the cellular interferon mediated antiviral response that in turn has been postulated to explain the corresponding clinical response. Clinical response to interferon-based regimens depends upon both viral factors (including NS5A and E2 glycoprotein) as well as host genetic factors, including lambda interferon polymorphisms [34], but the viral genotype assigned by clinical labs should closely reflect related strains and ideally indicate the historical antiviral response for those strains. Data from a chimeric mouse model, as well as anecdotal clinical data, suggests the RF1_2k/1b strain is more resistant to interferon than some genotype 2 strains [32]. As protease inhibitors and other directly acting antivirals become available, it will become increasingly important to know the genotype of each viral drug target of the isolate infecting the patient in order to determine the most effective therapy for that patient, and minimize the side-effects of therapy. Data from the PROVE 3 Protease Inhibitor trial [35], among others [18], suggests that subtyping may be clinically useful. Unfortunately, current methods for HCV genotyping primarily solely targeting the 5'UTR and possibly contiguous core (C) structural regions do not provide sufficient information across the entire genome to detect the possibility of recombinant species which may be critical for the determination for treatment efficacy.

In conclusion, we report here the first naturally occurring HCV recombinant in the United States. While clearly an independent event from other recombinants, this strain shares several characteristics with those previously reported in that it has genotype 2 5' UTR and structural genes, and a crossover point near the NS2/3 junction. At this time we cannot tell whether this recombinant strain is circulating in patients besides the one reported here, but the patient was viremic from this strain for months and likely years. Hybridization probe techniques and DNA sequencing targeting only the 5' UTR/core regions are frequently used to clinically genotype HCV to determine the dose and duration of therapy. One advantage of using direct DNA sequencing to genotype viruses is that the DNA sequence of amplified regions can be aligned with known recombinants, such as the strain reported here, particularly if multiple regions are sequenced. Using this approach, undiscovered recombinants may still be missed depending on the regions amplified, but at least an assessment of whether further testing is needed to rule out known recombinants can be made. The presence of circulating recombinants of HCV may have significant ramifications for the efficacy and selection of therapy. Clearly more comprehensive HCV genotyping is required to ascertain the significance of HCV recombinant isolates in clinical practice.

## Abbreviations

HCV: Hepatitis C Virus; UTR: untranslated region; FDA: Food and Drug Administration; CE: European Conformity.

## Competing interests

The authors declare that they have no competing interests.

## Authors' contributions

MAA and DB contributed equally to this work. MAA utilized consensus primers to perform RT-PCR amplifications, DNA sequencing analyses and alignment of both 5'UTR and NS5B regions in the identification of the recombinant HCV isolate and review of the manuscript. DB designed the primers and performed the RT-PCR amplification, cloning, DNA sequencing analysis and alignment of the 7 overlapping regions across the entire genome of the recombinant HCV isolate and review of the manuscript. IHA assisted DB in performing the RT-PCR amplification, cloning, sequencing analysis and sequencing alignment of the 7 overlapping regions across the entire genome of the recombinant HCV isolate. RS, a clinician researcher who manages patients infected with HCV at UWHC, supervised the project, assisted with data analysis and interpretation, and preparation of manuscript. WMR, Technical Director of the Molecular Diagnostics Clinical Laboratory at UWHC, supervised the project, assisted with data analysis and interpretation, and preparation of manuscript. All authors read and approved the final manuscript.
